# Hydrogen sulfide exposure induces NLRP3 inflammasome‐dependent IL‐1β and IL‐18 secretion in human mononuclear leukocytes *in vitro*

**DOI:** 10.1002/cre2.69

**Published:** 2017-07-03

**Authors:** Amina Basic, Sara Alizadehgharib, Gunnar Dahlén, Ulf Dahlgren

**Affiliations:** ^1^ Department of Oral Microbiology and Immunology Institute of Odontology, Sahlgrenska Academy, University of Gothenburg Sweden

**Keywords:** hydrogen sulfide, IL‐1ß, IL‐18, monocytes, NLRP3 inflammasome, periodontitis

## Abstract

The aim was to investigate if hydrogen sulfide (H_2_S) induces the formation of the NLRP3 inflammasome and subsequent IL‐1β and IL‐18 secretion in human peripheral blood mononuclear cells (PBMCs) and in the human monocyte cell line THP1. Bacterial production of H_2_S has been suggested to participate in the inflammatory host response in periodontitis pathogenesis. H_2_S is a toxic gas with pro‐inflammatory properties. It is produced by bacterial degradation of sulfur‐containing amino acids, for example, cysteine. We hypothesize that H_2_S affects the inflammatory host response by inducing formation of the NLRP3 inflammasome and thereby causes the secretion of IL‐1ß and IL‐18. PBMCs from eight healthy blood donors, the human monocyte cell line THP1 Null, and two variants of the THP1 cell line unable to form the NLRP3 inflammasome were cultured in the presence or absence of 1 mM sodium hydrosulfide (NaHS) in 24‐well plates at 37°C for 24 hr. Supernatants were collected and the IL‐1β and IL‐18 concentrations were measured with DuoSet ELISA Development kit. PBMCs exposed to NaHS produced more IL‐1ß and IL‐18 than unexposed control cells (*p* = .023 and *p* = .008, respectively). An increase of extracellular potassium ions (K^+^) inhibited the secretion of IL‐1ß and IL‐18 (*p* = .008). Further, NaHS triggered the secretion of IL‐1ß and IL‐18 in human THP1‐Null monocytes (*p* = .0006 and *p* = .002, respectively), while the NaHS‐dependent secretion was reduced in the monocyte cell lines unable to form the NLRP3 inflammasome. Hence, the results suggest that NaHS induces the formation of the NLRP3 inflammasome and thus the secretion of IL‐1ß and IL‐18. Enhanced NLRP3 inflammasome‐dependent secretion of IL‐1β and IL‐18 in human mononuclear leukocytes exposed to NaHS *in vitro* is reported. This may be a mode for H_2_S to contribute to the inflammatory host response and pathogenesis of periodontal disease.

## 1 INTRODUCTION

Periodontitis, a bacteria‐induced inflammation, is the result of a disruption of the homeostasis between the bacteria and the host. This dysbiosis is characterized by a high bacterial diversity and the growth of anaerobic, Gram‐negative, and proteolytic bacteria in the subgingival pocket (Kilian, Chapple, Hannig, et al., 2016). The new biofilm produces, among other metabolites, hydrogen sulfide (H_2_S), suggested to be part of the pathogenesis of periodontal disease because it is well known for its toxicity. H_2_S is produced by bacterial degradation of sulfur‐containing amino acids, for example, cysteine (Kuester & Williams, 1964) by many different bacterial strains (Basic, Blomqvist, Carlén, & Dahlén, 2015; Persson, Claesson, & Carlsson, 1989), and its presence has been reported in gingival crevicular fluid (Persson, 1992).

Although, it is generally accepted that the formation of the subgingival biofilm is essential for the induction of periodontitis, it remains unclear whether and how the biofilm and/or its end products contribute to the inflammatory host response that is characteristic for the disease. Monocytes/macrophages play an important role in the immune response of the host because they are able to produce a large variety of cytokines, for example, IL‐1β. The possible role of H_2_S on host cells in the periodontium is ambiguous because previous studies have reported both pro‐ and anti‐inflammatory properties of H_2_S (Whiteman & Winyard, 2011; Zhang & Bhatia, 2008). H_2_S can induce pro‐inflammatory cytokine IL‐8 production from gingival and oral epithelial cells *in vitro* (Chen, Kajiya, Giro, et al., 2010), but it has also been shown to cause cell death in lymphocytes and reduce their IL‐2 production. (Mirandola, Gobbi, Sponzilli, et al., 2007).

The pro‐inflammatory cytokine IL‐1β has been implicated as a key cytokine in periodontal disease (Graves, 2008; Kornman, 1997; Yilmaz & Lee, 2015). An inactive form of the protein (pro‐IL‐1β) is cleaved to its bioactive form, IL‐1β, via the formation of a multiprotein complex, called the NLRP3 inflammasome (Pétrilli et al., 2007). This is conducted via the cleavage and activation of pro‐caspase‐1 to its active form caspase‐1. In the same way as pro‐IL‐1β, pro‐IL‐18 is cleaved to IL‐18. A variety of substances, such as ATP, peptidoglycans, and crystals can induce the formation of the NRLP3 inflammasome and consequently the release of IL‐1β and IL‐18 (Pétrilli et al., 2007). A previous report has shown that NaHS also is able to induce the production and secretion of IL‐1β in the human monocyte cell line U937 (Zhi, Ang, Zhang, Moore, & Bhatia, 2007), but if this is mediated through the formation of the NLRP3 inflammasome remains unclear.

The aim of this study was to investigate the effect of hydrogen sulfide on the inflammatory response by studying human mononuclear leukocytes *in vitro* and their secretion of IL‐1β and IL‐18.

## 2 MATERIAL AND METHODS

### 2.1 Isolation of cells from human peripheral blood

Blood from eight and 10 unidentified donors respectively, collected at the Sahlgrenska University Hospital in Gothenburg, Sweden, was used in this study. The subjects that donate the blood consent that the blood may be used in research but no information of the subjects is available for the researchers. The subjects are defined as “healthy” because they fulfill the requirements to donate blood to the hospital blood bank, but the status of their oral health is not known. The peripheral blood mononuclear cells (PBMCs) were extracted by centrifugation using Ficoll‐Pague Plus (GE Healthcare Bio‐Sciences AB, Uppsala, Sweden). The cells were suspended in a solution containing Dulbecco´s Modified Eagle´s Medium, 5 % heat‐inactivated human AB serum (Sigma‐Aldrich Sweden AB, Stockholm Sweden), 100 U/mL penicillin, and 100 μg/mL of streptomycin (Invitrogen, Lidingö, Sweden). Using 0.4% Trypan Blue (Sigma‐Aldrich Sweden AB), the cell viability was determined, and the cells were counted in a Bürker chamber.

### 2.2 The human monocyte cell line THP1

The model cell line THP1‐Null (InvivoGen, San Diego, CA, USA) expresses high levels of NLRP3, ASC, and pro‐caspase‐1. In contrast, the ASC‐deficient THP1‐defASC cell line and the NLRP3‐deficient human monocytes THP1‐defNLRP3 (also InvivoGen) are both unable to form the NLRP3 inflammasome. The cells were cultured in RPMI 1640 growth medium (Invitrogen, Sweden) with 10% heat‐inactivated fetal bovine serum (Invitrogen, Sweden), 50 U/mL penicillin, and 50 μg/mL of streptomycin (Invitrogen, Sweden). The cell viability was determined using 0.4 % Trypan Blue, and the cells were counted.

### 2.3 H_2_S exposure

The cells (2 × 10^6^ PBMCs/THP1 cells/mL) were exposed to 1 μL/mL lipopolysaccharides (LPS, from Escherichia coli, Sigma‐Aldrich Sweden AB) for 3 hr at 37°C (humidified atmosphere, 5% CO_2_) in 96‐well plates to induce the production of pro‐IL‐1β. This step was, however, skipped for the last experiment without pre‐exposure to LPS. NaHS was used as H_2_S source, and the cells were exposed to 1 mM for 24 hr. In order to block the activation of the NLRP3 inflammasome, 130 mM of potassium chloride (KCl; Merck KGaA, Darmstadt, Germany) was added (Pétrilli et al., 2007). To test the method, 200 μg/mL aluminum potassium sulfate dodecahydrate (Merck) was used as a positive control. The cells exposed to NaHS were compared with unexposed controls.

### 2.4 The secretion of cytokines

The secretion of IL‐1β and IL‐18 by PBMCs/THP1 cells was measured with the use of the DuoSet ELISA Development Kit (R&D Systems, Abingdon, UK) according to the manufacturer´s instructions. Briefly, the plates were coated with capture antibody overnight. The supernatants were incubated with a cytokine‐specific biotinylated detection antibody and marked with streptavidin‐conjugated horseradish‐peroxidase. After the addition of substrate, the absorbance was recorded with an ELISA microplate reader (BioPlex 200 instrument equipped with BioManager analysis software (Bio‐Rad Laboratories AB, Solna, Sweden)).

### 2.5 Statistical analyses

Statistical analyses were performed in GraphPad Prism 6.0. The results from PBMCs were analyzed with Wilcoxon Signed‐Rank Test while the results from THP1 cells were compared using Mann–Whitney U Test.

## 3 RESULTS

### 3.1 Cytokine secretion by human peripheral blood leukocytes

**Figure 1:**
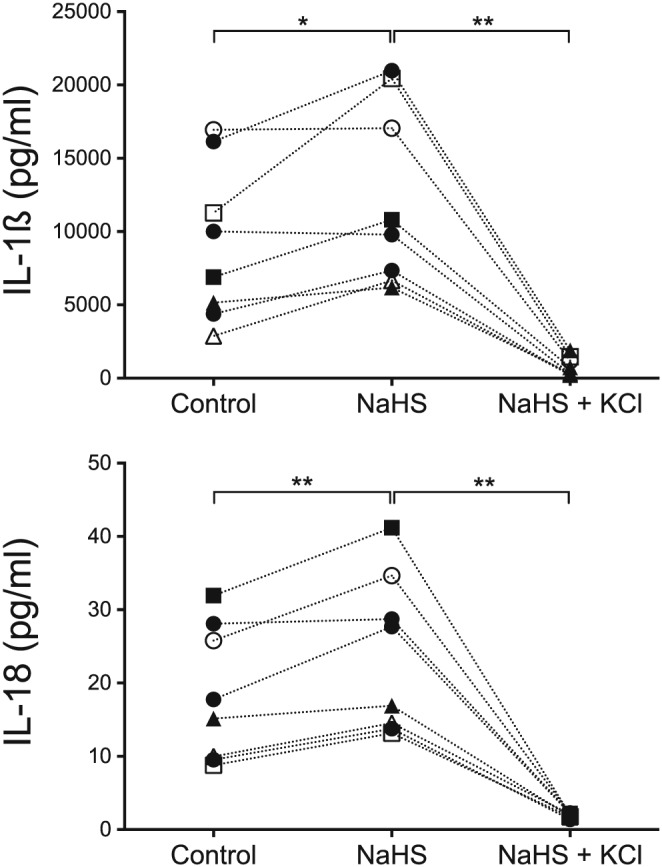
The IL‐1ß and IL‐18 secretion of peripheral blood mononuclear cells, measured from eight donors exposed to 1 mM hydrogen sulfide ions (NaHS) for 24 hr. The cells were pre‐exposed to lipopolysaccharides prior to NaHS. A Wilcoxon Signed‐Rank Test revealed a statistically significant difference (*p* = .023) in IL‐1ß secretion when the cells were exposed to NaHS compared with unexposed control cells. IL‐18 secretion was also statistically significant (*p* = .008). A statistical difference (*p* = .008 for both IL‐1ß and IL‐18) was also seen between the cells exposed to NaHS and KCl compared with only NaHS. The different symbols in the figures illustrate eight different blood donors

The secretion of IL‐1ß and IL‐18 after NaHS exposure compared with unexposed peripheral blood cells is seen in Figure 1 for eight blood donors. PBMCs exposed to NaHS produced significantly more IL‐1ß and IL‐18 (*p* = .023 and 0.008, respectively). The secretion of both IL‐1ß and IL‐18 (both *p* = .008) was inhibited when the extracellular concentration of KCl was increased.

### 3.2 Cytokine secretion from THP1 cells

**Figure 2:**
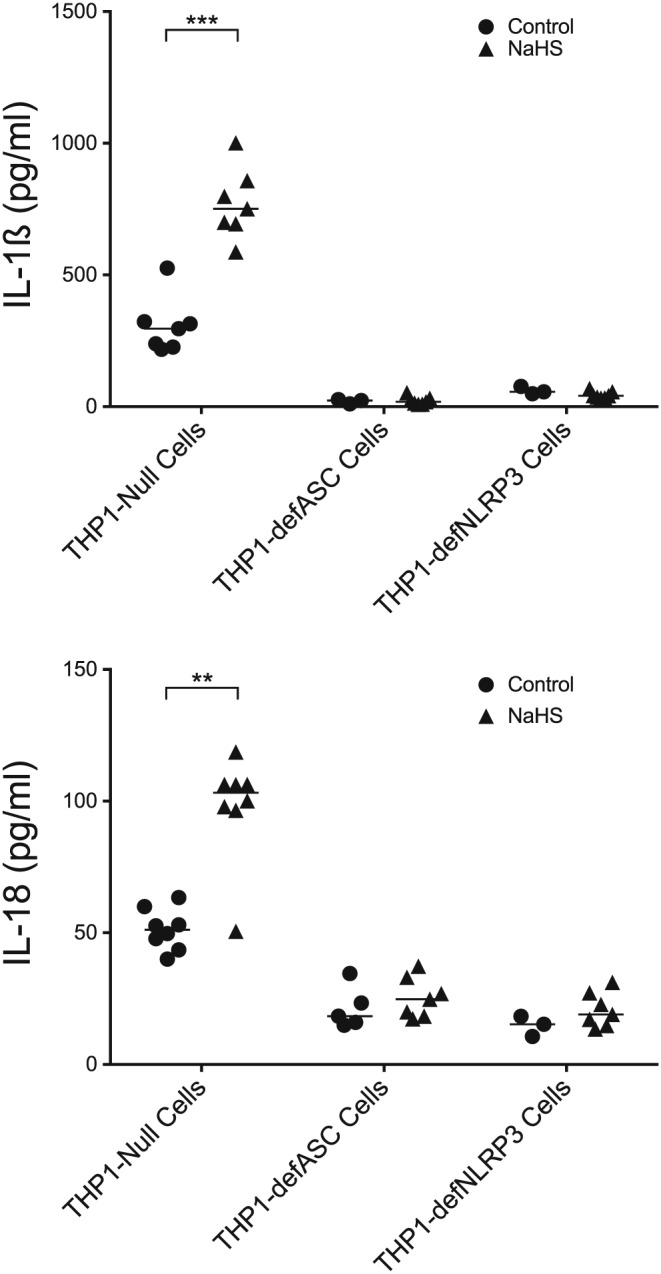
The IL‐1ß and IL‐18 secretion of three THP1 cell lines exposed to 1 mM NaHS for 24 hr. Prior to NaHS, the cells were exposed to lipopolysaccharides. The THP1‐Null cells showed a statistically higher IL‐1ß and IL‐18 secretion when exposed to NaHS (Mann–Whitney U test, *p* = .0006 for IL‐1ß and *p* = .002 for IL‐18) compared with unexposed cells. When the other two cell lines were tested, both unable to form the NLRP3‐inflammasome, there was no difference in IL‐1ß and IL‐18 secretion when exposed to NaHS compared to control. The median of the group is shown as a vertical line

The IL‐1ß secretion in human THP1 Null monocytes was triggered when the cells were exposed to NaHS (*p* = .0006) compared with unexposed cells (Figure 2). A similar result was seen for IL‐18 secretion (*p* = .002). The two monocyte cell lines that are unable to form the NLRP3 inflammasome, THP1‐defASC and THP1‐defNLRP3, did not produce more IL‐1ß and IL‐18 when exposed to NaHS than unexposed control cells.

### 3.3 Cytokine secretion from cells not exposed to lipopolysaccharides

**Figure 3:**
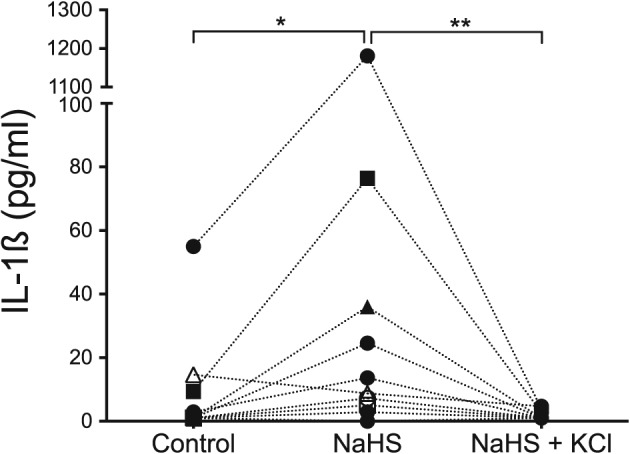
Peripheral blood mononuclear cells from 10 donors not pre‐exposed to lipopolysaccharides, but only exposed to 1 mM NaHS for 24 hr. The exposure to NaHS resulted in a statistically significant difference in IL‐1ß secretion compared with control cells (*p* = .020, Wilcoxon Signed‐Rank Test). A reduction in IL‐1ß secretion was seen when the cells were exposed to increased extracellular KCl concentrations (*p* = .004)

The IL‐1ß secretion was also measured from cells not pre‐exposed to LPS. Human PBMCs from 10 donors were exposed to NaHS (Figure 3). A significantly higher IL‐1ß secretion was seen when the cells were exposed to NaHS compared with unexposed control cells (*p* = .020) or when KCl was added (*p* = .004).

Cellular viability was also assessed after 24 hr of incubation. The results showed similar viability for cells exposed to NaHS (88 ± 4%) as for unexposed control cells (92 ± 3%).

## 4 DISCUSSION

This study investigated if NaHS induced the formation of the NLRP3 inflammasome and thus subsequent secretion of IL‐1β and IL‐18 in human peripheral blood mononuclear cells and in the human monocyte cell line THP1. An enhanced NLRP3 inflammasome‐dependent secretion of IL‐1β and IL‐18 in human mononuclear leukocytes exposed to NaHS *in vitro* was found.

The production and secretion of IL‐1β and IL‐18 is conducted as a result of two signals. The first signal stimulates the production of pro‐IL‐1β and pro‐IL‐18 in the nucleus and can be induced by LPS. The second signal, investigated in this study with the use of NaHS, leads to the formation of the NLRP3 inflammasome and thereby causes the release of IL‐1β and IL‐18 from the cell.

We also tested the effect of NaHS on IL‐1β secretion in PBMCs without pre‐exposure to LPS, to study if H_2_S could contribute to both production and secretion of the cytokines (Figure 3). The cells responded similarly to the cells that were pre‐exposed to LPS, but the secretion was lower than when the cells were pre‐exposed to LPS before adding NaHS. A reduced IL‐1β secretion without LPS treatment is in agreement with a previous report where ragweed pollen extract exposure in THP1 macrophages was studied (Varga et al., 2013). A previous study showed that NaHS exposure increased Akt phosphorylation, regulated by PI3K (Cai et al., 2007). This was confirmed in endothelial cells along with phosphorylation of ERK and p38 (Altaany, Yang, & Wang, 2013; Papapetropoulos, Pyriochou, Altaany, et al., 2009). Further, another study disclosed that exposure to NaHS, though the (ERK)‐NF‐κB signaling pathway, contributed to the production of IL‐1β (Zhi et al., 2007). This was conducted by IkBα degradation and consequently NF‐κB p65 activation. The study reported that the activation was highest after 30 min of incubation and at the concentration of 0.1 mM NaHS. There are, however, other reports claiming the opposite, that NaHS does not activate NF‐kB (Sulen, Gullaksen, Bader, et al., 2016; Whiteman et al., 2010). Nevertheless, it seems that H_2_S may substitute the LPS signal 1, possibly via mitogen‐activated protein kinases.

THP1 cells were used to investigate if the secretion of IL‐1β and IL‐18 was mediated through the formation of the NLRP3 inflammasome. The results showed a statistically significant increase in cytokine secretion in the THP1‐Null cell line when the cells were exposed to NaHS, illustrating that monocytes do secrete IL‐1β and IL‐18 in the presence of NaHS (Figure 2). However, two variants of the THP1 cell line that are unable to form the NLRP3 inflammasome did not produce IL‐1β and IL‐18 upon exposure to NaHS in our study. This strongly suggests that the secretion of the cytokines was mediated through the formation of the NLRP3 inflammasome.

Many different substances such as ATP, peptidoglycans, nigericin, or monosodium urate crystals may induce the second signal that activates the NLRP3 inflammasome, but the mechanism involved is still unknown. Pétrilli and coworkers (Pétrilli et al., 2007) suggested that the substances that activate the NLRP3 inflammasome have the ability to lower the intracellular potassium ion (K^+^) concentration, because the effect of all of the different substances are blocked if the efflux of K^+^ is inhibited. In our study, the cytokine production was inhibited when the extracellular concentration of K^+^ was increased. A previous report on porcine and bovine retinae showed the inhibitory effect of H_2_S donors on K^+^‐evoked [^3^H] D‐aspartate release (Opere, Monjok, Kulkarni, Njie, & Ohia, 2009). Furthermore, a study showed an inhibitory effect of H_2_S on potassium channels of trigeminal ganglion neurons (Feng, Zhou, Meng, et al., 2013) and another the effect of H_2_S on K_v_ 7.4 channels that resulted in vasodilation (Martelli, Testai, Breschi, et al., 2013). All these studies together suggest that the triggers of the NLRP3 inflammasome, including H_2_S, reduce the intracellular K^+^ concentration and thereby cause the formation of the NLRP3 inflammasome.

**Figure 4:**
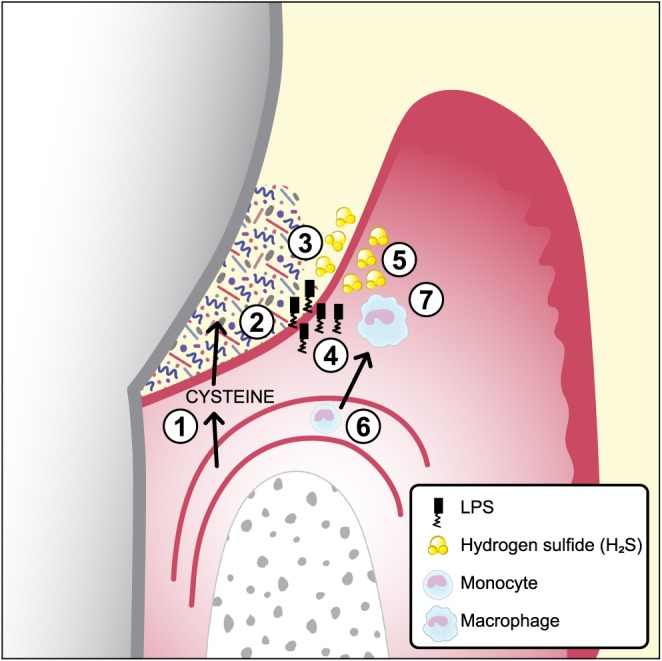
A schematic figure of an inflamed gingival pocket with subgingival plaque (biofilm) and monocytes/macrophages
Serum exudate from blood vessels containing serum proteins, peptides, and amino acids including cysteine.The exudate (gingival crevicular fluid) continues through the thin pocket epithelium (junctional epithelium) into the subgingival pocket.The subgingival plaque, containing numerous, mainly Gram‐negative, anaerobic bacteria with proteolytic capacity, degrade proteins, peptides, and amino acids including cysteine.Growing Gram‐negative anaerobes release lipopolysaccharides that penetrate the junctional epithelium into gingival connective tissues.Growing Gram‐negative anaerobes (*Fusobacterium* spp., *Porphyromonas gingivalis*, *Treponema* spp., and others) produce metabolites, for example, hydrogen sulfide (H_2_S).The inflammatory lesion attracts monocytes that migrate into the connective tissue and differentiate to macrophages.The effect of lipopolysaccharides and H_2_S on macrophages and the subsequent secretion of the pro‐inflammatory cytokines IL‐1β and IL‐18

Despite that it is generally acknowledged that H_2_S is a toxic gas, our understanding of its functions is poor. It is proposed that H_2_S has the ability to split essential disulfide bonds in proteins and can bind to metal ions (Beauchamp, Bus, & Popp, 1984). Further, H_2_S has been suggested to inhibit cytochrome oxidase (Nicholls & Kim, 1982), catalase, and myeloperoxidase (Claesson, Granlund‐Edstedt, Persson, & Carlsson, 1989). It has also been shown to induce apoptosis in human gingival fibroblasts *in vitro* (Zhang, Dong, & Chu, 2010). These factors are believed to explain the toxicity of H_2_S. Our study suggests one more way by which H_2_S may affect the host cells and thereby contribute to disease development (Figure 4). H_2_S may induce and/or maintain the host immune response by the secretion of the pro‐inflammatory cytokines IL‐1β and IL‐18 in macrophages. This may play an important role in the pathogenesis of periodontal disease, characterized by a proteolytic biofilm in the periodontal pocket.

The elevated secretion of IL‐1β and IL‐18 when exposed to NaHS shows the pro‐inflammatory capacity of H_2_S on human PBMCs from healthy blood donors (Figures 1 and 3). Because the cells were not separated further, it is unclear whether the production came from monocytes and/or lymphocytes. The ratio between these two was not further investigated in this study. Despite the small sample size of eight and 10 blood donors, a statistically significant increase of IL‐1β and IL‐18 secretion was seen in the presence of NaHS. However, there were distinct individual variations between the donors, which suggests that the sensitivity to NaHS exposure among the donors varies. Because the periodontal status of the donors is unknown, we can only speculate that the cells with higher IL‐1β and IL‐18 responses are from donors more prone to develop periodontal disease. This aspect is investigated in an ongoing clinical trial on periodontitis patients and healthy controls.

## 5 CONCLUSION

The production of pro‐inflammatory cytokines is essential in the induction and development of inflammatory diseases such as periodontal disease. We have studied the effect of the bacterial waste product H_2_S, by the use of NaHS, on cytokine secretion *in vitro*. Our results show higher secretion of IL‐1ß and IL‐18 from mononuclear leukocytes and THP1 cells when exposed to NaHS compared with unexposed controls. The NLRP3 inflammasome is essential for NaHS induced IL‐1ß and IL‐18 secretion in monocytes. The results of our study suggest a possible model (Figure 4) on how bacterial activity, through the end products of the biofilm, may contribute to the inflammatory host response and disease development. Further *in vivo* studies are needed to examine the function of H_2_S in the pathogenesis of periodontal disease.
